# Parent-adolescent discrepancies in educational expectations, relationship quality, and study engagement: a multi-informant study using response surface analysis

**DOI:** 10.3389/fpsyg.2024.1288644

**Published:** 2024-03-21

**Authors:** Youzhi Song, Jianjun Wu, Zongkui Zhou, Yuan Tian, Weina Li, Heping Xie

**Affiliations:** ^1^Key Laboratory of Adolescent Cyberpsychology and Behavior (CCNU), Ministry of Education, Wuhan, China; ^2^School of Psychology, Central China Normal University, Wuhan, China; ^3^Wuhan South Lake Middle School, Wuhan, China; ^4^Department of Management, Hunan Policy Academy, Changsha, China; ^5^School of Fundamental Education, South China Normal University, Shanwei, China

**Keywords:** parent-adolescent educational expectations, discrepancies, polynomial regressions, relationship quality, response surface analysis, study engagement

## Abstract

Whether parental educational expectations for adolescents serve as a source of motivation or stress depends on the extent to which adolescents hold expectations for themselves. Previous research on the discrepancies between parental and adolescent educational expectations and their impact on learning engagement has been limited by traditional statistical tests, and lacking an examination of the internal mediating mechanism of parent–child relational quality from both parental and adolescent perspectives. This cross-sectional study, utilizing a multi-informant design, examined the association between discrepancies in parents’ and adolescents’ reports of expectations, and adolescents’ study engagement, as well as the mediating role of parent–child relational qualities perceived by both parties. The sample for this study consisted of 455 adolescents and their parents from 10 classes in a junior high school in Wuhan, Hubei Province, China. The adolescents had an average age of 12.8 years, and 51.6% of them were boys. Both parents and adolescents reported on their expectations and perceived relational quality, while adolescents also filled out questionnaires assessing their learning engagement. Data were analyzed using polynomial regressions with response surface analysis. The results revealed that when adolescents reported high expectations, regardless of whether their parents reported high or low expectations, adolescents reported satisfied relationships and high learning engagement. In contrast, parents reported satisfied relationships when both parties reported high expectations, or when parents reported higher expectations than adolescents. Lastly, the association between discrepancies in expectations and learning engagement was significantly mediated by adolescent-reported relationships but not parent-reported ones. These findings highlight the importance of considering multiple perspectives when studying the association between expectations and adolescent study engagement. This research advances our comprehension of the dynamics between parent-adolescent educational expectation discrepancies and adolescent learning engagement, offering insights for more nuanced and effective parenting strategies tailored to foster optimal educational outcomes.

## 1 Introduction

Parents have long held high expectations for their children’s future success (Pinquart and Ebeling, 2020). Parental educational expectations refer to parents’ realistic predictions about their children’s academic outcomes, such as course grades, skill development, and the highest level of education attained (Yamamoto and Holloway, 2010). Parental educational expectations have been found to be beneficial in fostering adolescents’ learning engagement and academic progress (Pinquart and Ebeling, 2020). However, when parental educational expectations are excessive, they can cause stress for their children (Tan and Yates, 2011).

Academic-related stress is a pervasive issue among adolescents, with particularly acute manifestations in China, a society steeped in Confucian cultural traditions (Tan and Yates, 2011). This cultural backdrop places a premium on academic achievement, viewed not only as a personal accomplishment but also as a critical component of filial duty that enhances family honor. Conversely, academic shortcomings are perceived as failing to uphold family dignity, propelling adolescents into an arduous journey to meet or exceed their parents’ high educational expectations (Tan and Yates, 2011). Such dynamics, emblematic of the Confucian valorization of education, may lead to a range of negative outcomes, including academic burnout, anxiety, and in severe cases, disengagement from the educational process (Peleg et al., 2016; Ribeiro et al., 2018; Pascoe et al., 2020). This study is specifically designed to delve into the relationship between the educational expectations discrepancies among Chinese adolescents and their parents and the adolescents’ engagement in learning. Furthermore, it investigates the mediating role of perceived parent–child relationship quality from both perspectives. By focusing on this demographic within the context of Confucian cultural influence, we aim to contribute to a nuanced understanding of how educational expectations within such a cultural framework relate to student engagement and the perception of parent–child relationship quality.

Previous research has indicated that adolescents who perceive their parents’ expectations to be higher than their own tend to have lower academic self-efficacy and future achievement. Conversely, adolescents who perceive their parents’ expectations to be lower than their own tend to have a higher future achievement (Wang and Benner, 2014; Lv et al., 2018). Whether parental educational expectations serve as motivators or barriers can depend on the youth’s own educational expectations. In other words, a youth’s own level of educational expectations may serve as a benchmark for determining whether parental expectations are excessive. While previous research has laid a solid foundation for investigating the role of discrepancies in parent–child educational expectations (Pinquart and Ebeling, 2020), there are still some issues that require further investigation. First, in terms of statistical testing methods, previous research on discrepancies in parent–child educational expectations has often examined correlations between difference scores (e.g., algebraic, squared, or absolute difference between two scores) and the outcome variable (Wang and Benner, 2014; Lv et al., 2018). However, the “difference scores” method has several limitations, such as reduced dimensionality of variables, difficult interpretation of coefficients, lack of parameter restrictions, and decreased reliability (Shanock et al., 2010). These limitations can be overcome through polynomial regression with response surface analysis, which is specially developed to address variable matching and discrepancy (Edwards and Cable, 2009; Shanock et al., 2010; Schönbrodt et al., 2018). Therefore, this study aimed to apply polynomial regression and response surface analysis to conduct a more nuanced examination of the congruence and incongruence between adolescents’ and parents’ educational expectations. Second, to our knowledge, there is limited research exploring the internal mediation mechanisms between the discrepancies in parent-adolescent education expectations and adolescent academic behaviors from the perspective of family member relationships. The parent-adolescent relationship, a vital social family resource for youth and adolescents, plays a critical role in their learning and development (Coleman, 1988; Havermans et al., 2014). It may serve as a mediator in the relationship between parent-adolescent discrepancies in educational expectations and academic behaviors. Therefore, this study aimed to investigate the associations between parent-adolescent discrepancies in educational expectations and adolescents’ study engagement, as well as the mediating roles of the quality of parent–child relationships. The current research can contribute to a better understanding of how discrepancies in parent-adolescent educational expectations function in adolescent learning and provide more effective and targeted recommendations for parenting practices.

Study engagement is a persistent, positive, fulfilling, study-related state of mind characterized by vigor, absorption, and dedication (Schaufeli et al., 2002). According to the definition of study engagement, a highly engaged learner is someone who has abundant energy and flexibility, a strong focus and interest, and a sense of meaning and challenge in the learning process (Schaufeli et al., 2002; Salmela-Aro and Upadaya, 2012). Previous studies have found that study engagement predicts the learning process and academic achievement and is also a crucial indicator of healthy student development (Upadyaya and Salmela-Aro, 2013; Martins et al., 2022). The self-system model of motivational development (SSMMD) developed by Skinner et al. (2008) provides a theoretical foundation for linking parent-adolescent discrepancies in educational expectations to adolescents’ study engagement. The SSMMD depicts contextual predictors of students’ social interactions with family, peers, and teachers that influence students’ engagement in learning by acting upon their self-systems that are organized around three basic psychological needs: competence, autonomy, and relatedness (Ryan and Deci, 2000; Skinner et al., 2008). Therefore, the study employs the SSMMD to primarily elucidate and substantiate the relationship between parent-adolescent discrepancies in educational expectations and adolescent study engagement.

According to SSMMD, adolescents who hold higher educational expectations than their parents are likely to have a high degree of autonomy and, thus, intrinsic motivation in their academic pursuits (Zhen et al., 2017). Intrinsically motivated adolescents are more likely to remain highly focused and persistent in their learning, experiencing pleasure and value in their educational pursuits (Karimi and Sotoodeh, 2020). Conversely, when adolescents’ educational expectations fall below their parents’ expectations, adolescents may be pushed forward academically by their parents, and their need for autonomy is likely to be frustrated; they may become less engaged and more likely to avoid or give up when faced with academic challenges (Agliata and Renk, 2008). Additionally, adolescents with lower educational expectations may experience frustrated competence due to their inability to meet their parents’ overly high educational expectations. Both thwarted needs for autonomy and competence can seriously undermine youths’ intrinsic motivation, reducing engagement and causing them to feel academically passive and helpless (Karimi and Sotoodeh, 2020). Parent-adolescent congruent educational expectations can be further divided into two types: one in which both adolescents and parents have congruent and high educational expectations, and the other in which they have congruent but low educational expectations. Although neither type thwarts the child’s need for autonomy, there may be substantial differences in their impact on adolescent study engagement. The former type is more likely to create a converging force of intrinsic and extrinsic motivation that promotes adolescent engagement in learning. In contrast, the latter is permissive and indulgent, and adolescents in this situation will likely show minimal study engagement.

Parent–child relationships are innate and one of the children’s earliest and most important social contacts (Laursen and Collins, 2009). Wu et al. (2011) identified several essential components that constitute a high-quality parent–child relationship, such as understanding and communication, low excoriation and control, liking and respect, and growth and tolerance. Social family resource theory suggests that the parent–child relationship is a crucial family social resource for adolescents that plays a vital role in their learning and development (Coleman, 1988). Adolescents who spend more time with their parents and have higher-quality relationships are more likely to receive academic support and guidance from their parents (Coleman, 1988; Mo and Singh, 2008). Conversely, less parental presence and poorer parent–child relationships can negatively affect children’s learning and growth (Popov and Ilesanmi, 2015). In addition, relationship needs are closely related to individuals’ intrinsic motivation from the perspective of basic psychological needs (Ryan and Deci, 2000; Zhen et al., 2017). An environment with a higher sense of security and belonging will stimulate more intrinsically motivated behaviors. In the broader theoretical framework proposed by Skinner et al. (2008), a good parent–child relationship provides a warm and harmonious family atmosphere and a conducive learning environment for adolescents. Therefore, adolescents in good parent–child relationships exhibit more enthusiasm, curiosity, and interest in academics and are more committed to learning (Wu and Yang, 2012). Empirical studies have found that parent–child relationship quality significantly predicts children and adolescents’ life satisfaction, academic engagement, academic competence, and scores on standardized achievement tests (Murray, 2009; Jiménez-Iglesias et al., 2017; Markkula et al., 2021; Li et al., 2022). Conversely, families with divorced parents have more parental conflict and poorer quality of parent–child relationships, leaving children with fewer family social resources and ultimately hindering their academic engagement (Havermans et al., 2014).

Self-determination theory is a macro theory of human motivation and personality that deals with people’s inherent growth tendencies and innate psychological needs. The Relationship Motivation Theory within the Self-Determination Theory aids in elucidating the relationship between educational expectations and the quality of parent–child relationships. This theory underscores the significance of supportive significant others in meeting an individual’s needs for autonomy, competence, and relatedness (La Guardia and Patrick, 2008). When these needs are satisfied, individuals experience higher self-esteem, vitality, positive affect, relationship satisfaction, and commitment. Conversely, when partners are excessively controlling, have unreasonable expectations, or are overly challenging or rejecting, optimal functioning is compromised (La Guardia and Patrick, 2008; Deci and Ryan, 2014). According to the Relationship Motivation Theory, adolescents who hold higher educational expectations than their parents tend to have more autonomy, which is positively correlated with their happiness and positive behavior (Deci and Ryan, 2000). This situation can lead to more positive parent–child relationships (Fredrickson, 2001). Conversely, when adolescents have lower educational expectations than their parents, their autonomy and competence are undermined, resulting in negative emotions such as anxiety and depression (Deci and Ryan, 2000; Vansteenkiste and Ryan, 2013). This situation increases the likelihood of parent–child conflict and can damage the quality of the relationship. When adolescents and their parents have congruent and high educational expectations, the matching expectations contribute to a good parent–child relationship. Conversely, when expectations are low on both sides, the level of connection between child and parent tends to be lower and the relationship quality poorer.

It is worth noting that as individual autonomy and self-awareness continue to increase in adolescence, young people’s needs become more complex and varied (Smetana et al., 2006), leading to discrepancies between adolescents’ and parents’ perceptions of their relationship (Eccles et al., 1993; Nelemans et al., 2016). What parents perceive as a good parent–child relationship may not align with what adolescents need or want. Early research has indicated that due to the potential influence of social desirability bias, parents may tend to report what they perceive as ideal parenting behaviors, thereby overestimating the warmth and control they exhibit (Bögels and Van Melick, 2004; Waylen et al., 2008). Further, children’s perceptions of parenting are more likely to impact their affect and behavior than parents’ perceptions of parenting (Janssen et al., 2021). Therefore, it can be hypothesized that adolescents’ perceptions of the parent–child relationship, rather than parental perceptions, may significantly mediate the association between parent–child discrepancies in educational expectations and study engagement.

In sum, the current study proposed the following research hypotheses: (1) There are congruent effects of educational expectations between parents and adolescents, indicating that adolescents demonstrate higher levels of engagement in learning when both adolescents and parents report congruent and higher educational expectations. (2) Additionally, higher quality parent–child relationships are reported by both parents and adolescents when both adolescents and parents report congruent and higher educational expectations. (3) The incongruent effects of educational expectations would suggest that when adolescents hold higher levels of educational expectations than what is reported by their parents, they are notably more likely to report higher levels of engagement in learning and better parent–child relationships. (4) Conversely, parents report better relationship quality when they hold higher educational expectations than their adolescents. (5) Adolescents’ reports of the parent–child relationship, rather than parents’, may mediate the association between congruence and incongruence in adolescents’ versus parents’ reports of educational expectations and study engagement.

## 2 Methods

### 2.1 Sample and procedure

This research collected multi-informant data from parents and teenagers. Using a cluster random sampling approach, we surveyed 613 junior high school students and 519 parents from 10 classes at a junior high school in Wuhan, Hubei Province, China. After thoroughly matching adolescents and their parents, 455 parents-adolescent dyads comprised the total sample for our analyses. The adolescents (51.6% boys) had a mean age of 12.8 (SD = 0.67) years, with 240 (52.7%) students in grade 7 and 215 (47.3%) students in grade 8. Youth from intact families accounted for 92.5% of the sample, whereas those from divorced, remarried, and single-parent households accounted for 4.6, 2.2, and 0.5%, respectively. The mothers and fathers comprise 371 and 84 individuals, with average ages of 41.01 (SD = 3.56) and 44.38 (SD = 4.79), respectively. Regarding paternal education, 14.3% have a high school diploma or less, 27.4% have received specialized education, 41.7% hold a bachelor’s degree, and 16.7% have a master’s degree or higher. Concerning maternal education, 16.4% have a high school diploma or less, 23.7% have received specialized education, 48.2% hold a bachelor’s degree, and 11.6% have a master’s degree or higher.

Student data were collected in the school’s computer classroom using Sojump, a popular online survey platform in China (similar to SurveyMonkey in the United States). A trained research assistant oversaw the entire administrative process, while a computer teacher was responsible for technical concerns and maintaining discipline. Informed consent was obtained from the adolescents. After obtaining informed parental consent, we gathered parent data online by releasing a link to the Sojump survey in a WeChat group for parents that had previously been created. The research was approved by the ethics committee.

### 2.2 Measures

#### 2.2.1 Educational expectations

The study used the educational expectations scale developed by Wang and Liu (2005) to assess the educational expectations of adolescents and their parents. They found a close association between discrepancies in educational expectations among junior high school students and their parents and the quality of parent–child relationships through their self-developed education expectation scale (Wang and Liu, 2005). The scale consisted of eight items that evaluated parents’ and teenagers’ expectations regarding educational attainment (e.g., “Getting into a satisfactory college”), grades (e.g., “Higher test scores”), in-class performance (e.g., “Actively speak and ask questions in class” and “Attentive in class”), academic competence (e.g., “Highly efficient learning”), and others. Participants rated each item on a 4-point scale ranging from 1 (no expectation) to 4 (highest expectation), with higher scores indicating higher educational expectations. Previous studies (Liu, 2020) demonstrated high reliability, and in the present study, Cronbach’s alpha coefficients for adolescent and parental educational expectations were 0.91 and 0.93, respectively, indicating excellent reliability.

#### 2.2.2 Parent–child relationship

The study also used the parent–child relationship questionnaire developed by Wu et al. (2011) to separately assess adolescent- and parent-reported relationship quality. The questionnaire comprised 26 terms across four subscales: understanding and communication (e.g., “When I felt aggrieved, I was willing to tell my parents,” “When my child felt aggrieved, (s)he was willing to talk about it with me”), excoriation and controlling (reverse coding) (e.g., “When I disobeyed my parents or did not do what they say, I would be severely scolded by them,” “When my child disobeyed me or did not do what I say, (s)he would be severely scolded by me”), liking and respect (e.g., “When I talked, my parents listened patiently and attentively,” “I could listen patiently and attentively when my child was talking”), and growth and tolerance (e.g., “When there was a conflict between parents and children, the parents did not think it was necessarily my fault.,” “When there was conflict between parents and children, I did not think it was necessarily the child’s fault.”). Participants rated each term on a 5-point Likert scale ranging from 1 (completely untrue) to 5 (completely true), with higher scores indicating better parent–child relationship quality as perceived by adolescents or parents. This scale was effectively validated in Wu et al.’s (2022) study. The Cronbach’s alpha coefficients for adolescent and parent questionnaires were 0.95 and 0.92, respectively.

#### 2.2.3 Learning engagement

The Chinese version of the Utrecht Work Engagement Scale-student, revised by Fang et al. (2008), was employed to measure adolescents’ academic engagement. The scale consisted of 17 items measuring three subscales: vigor (e.g., “When I am studying, I feel mentally strong”), dedication (e.g., “My studies inspire me”), and absorption (e.g., “When I am studying, I forget everything else around me”). Participants rated the items on a 7-point Likert scale ranging from 1 (never) to 7 (always). Fang and Ding’s (2020) study confirmed the reliability of this scale in the Chinese population. The scale demonstrated good reliability in the present study (α = 0.96).

### 2.3 Data analysis

The present study employed polynomial regression with response surface analysis to model and visualize complex interactions and discrepancies in a three-dimensional space, offering a more intuitive and comprehensive understanding of how these discrepancies relate to adolescent learning engagement (Edwards and Cable, 2009). Data analysis was performed according to the procedures outlined by Barranti et al. (2017) using the RSA package (Schönbrodt and Humberg, 2023) in R 4.1.2. First, we examined the frequency of difference observations between adolescent and parent educational expectations, where a difference of more than half a standard deviation indicated a discrepancy (Shanock et al., 2010). Second, we standardized adolescent and parent reports of educational expectations using pooled standard deviations to ensure commensurate scaling and a shared midpoint (e.g., Van Petegem et al., 2020). Third, we conducted a series of polynomial regression analyses, regressing outcome variables on adolescent and parent-reported educational expectations, their squared terms, and their interaction term. Specifically, we used adolescents’ reports of parent–child relationship (model 1), parents’ reports of parent–child relationship (model 2), and study engagement (model 3) as dependent variables for the three polynomial regression models. Fourth, we examined the mediating roles of parent–child relationships in the model by adding adolescent and parent-reported parent–child relationships as predictor variables to model 3, constructing model 4 and model 5, respectively.

**Figure 1:**
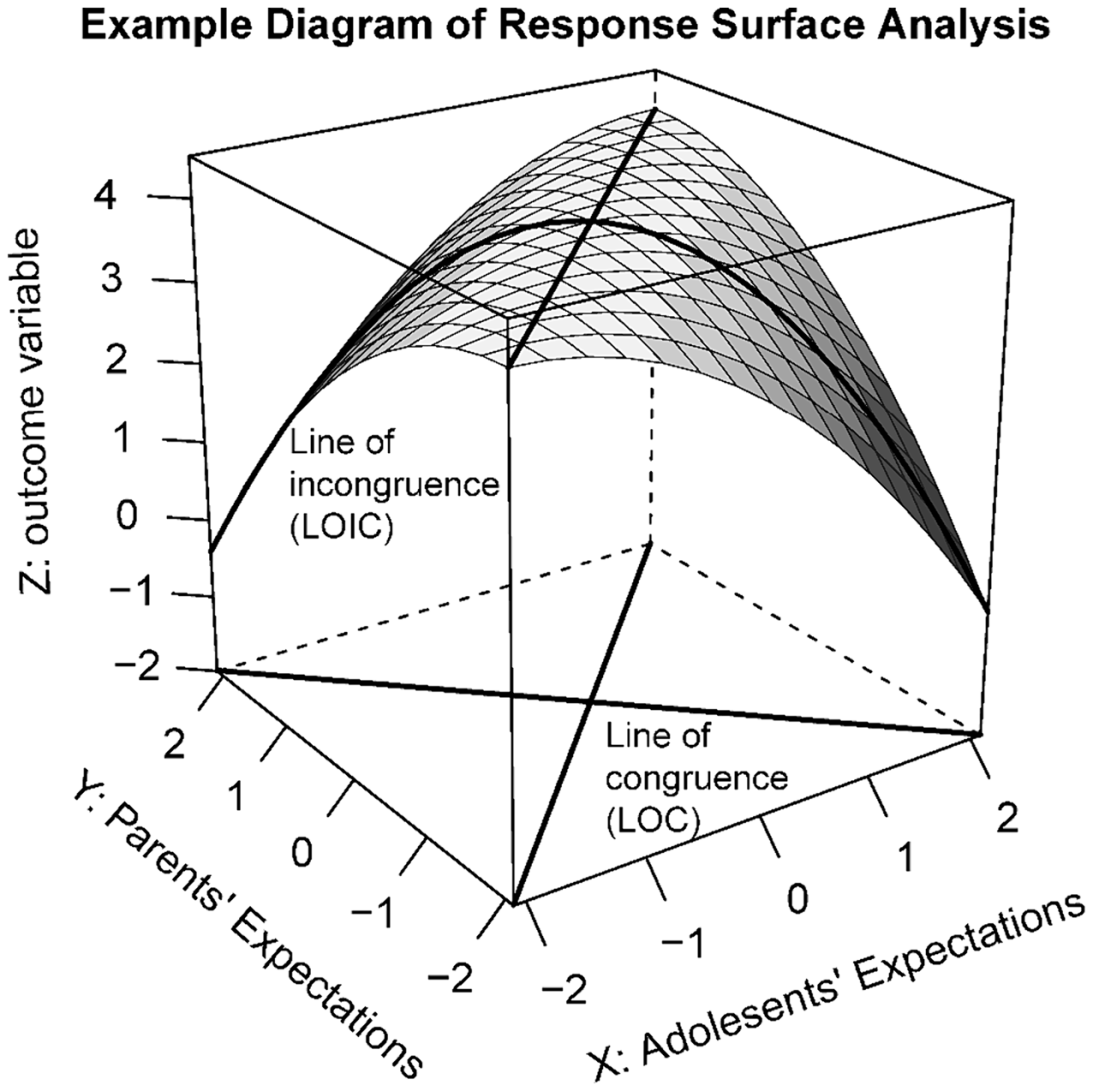
Example diagram of response surface analysis. Refer to Schönbrodt et al. (2018) for the response surface of the estimated regression equation Z = 4 + 0X + 0Y − 0.25×2 + 0.5XY − 0.25Y2.

Fifth, we used response surface analyses to interpret the results from the polynomial regression analyses (Schönbrodt et al., 2018). Response surfaces are a visual presentation of polynomial regression. It aids in visually depicting the three-dimensional distribution map of outcome variables, as illustrated in Figure 1. Line of Congruence (LOC) and Line of Incongruence (LOIC) adequately demonstrate the varied matching of education expectations between adolescents and parents. As shown in Figure 1, LOC refers to congruent education expectations between both parties. Along the LOC, moving from the outer to the inner direction, parent-adolescent education expectations consistently align, with values gradually increasing. LOIC signifies divergent education expectations between both parties. The left half of LOIC represents parents’ education expectations being higher than adolescents, while the right half is the opposite. The slopes and curvatures along the LOC and LOIC reflect the shape and trends of the response surface of the outcome variable in the three-dimensional coordinate system (Schönbrodt et al., 2018).

Finally, to ensure the robustness of the results, we used the “block variable” approach (Edwards and Cable, 2009) and bias-corrected confidence intervals constructed from estimates based on 10,000 bootstrap samples to test for the mediating roles (MacKinnon et al., 2004).

## 3 Results

### 3.1 Preliminary analyses

The means, standard deviations, and correlations of the study variables are presented in Table 1. There was no significant difference in educational expectations between adolescents and parents, as evidenced by the mean scores (*t*_(454)_ = 1.61, *p* = 0.123). However, using a cut-off point of 0.5 standard deviations, the results revealed that 61% of parent-adolescent dyads had discrepancies in educational expectations. In contrast, 39% of adolescents reported similar scores to their parents. Of these informant discrepancies, 34% of adolescents reported higher educational expectations, and 27% reported lower expectations than their parents. The descriptive analyses showed several observations with discrepant values, indicating the importance of examining the link between congruence and incongruence in educational expectations, parent–child relationships, and study engagement.

**Table 1:** Means, standard deviations, and correlations among the study variables (*n* = 455).

Variables	1	2	3	4	5	6	7
1. Gender	1						
2. Grade	−0.04	1					
3. AEE	−0.02	−0.15^***^	1				
4. PEE	0.02	−0.14^**^	0.17^***^	1			
5. ARR	0.02	−0.20^***^	0.31^***^	0.04	1		
6. PRR	−0.07	−0.13^**^	0.12^*^	0.24^***^	0.16^***^	1	
7. Learning Engagement	−0.02	−0.18^***^	0.52^***^	0.11^*^	0.56^***^	0.13^**^	1
*M*	0.52	0.47	3.63	3.59	3.80	3.57	5.53
SD	0.50	0.50	0.52	0.47	0.77	0.28	1.17

*n*, sample size; M, Mean; SD, Standard Deviation. ^*^p < 0.05; ^**^p < 0.01; ^***^p < 0.001.

Gender was dummy-coded, with 0 representing girls and 1 representing boys. Grades were dummy-coded, with 0 representing grade 7 and 1 representing grade 8. AEE refers to adolescents’ educational expectations; PEE refers to parents’ educational expectations; ARR refers to adolescent-reported parent–child relationships; PRR refers to parent-reported parent–child relationships.

Table 1 also showed a significant positive correlation between adolescent- and parent-reported educational expectations. Further, significant two-by-two correlations were observed between adolescent-reported educational expectations, adolescent-reported parent–child relationship, parent-reported parent–child relationship, and learning engagement. Parent-reported educational expectations were significantly associated with the parent-reported parent–child relationship but not the adolescent-reported parent–child relationship. Additionally, adolescents’ grade levels were significantly related to the outcome variables, with eighth-graders reporting lower levels of parent–child relationships and learning engagement than seventh-graders. No significant associations were found between adolescents’ gender and any outcome variables. Therefore, we controlled for adolescents’ grade levels in subsequent analyses.

### 3.2 Polynomial regression on parent–child discrepancies in educational expectations

In this section, we first presented the polynomial regression and response surface analysis of the parent-adolescent relationship quality reported by both parties on discrepancies in parent–child educational expectations. Next, we reported, without considering the mediating variables, the polynomial regression and response surface analysis of the study engagement on parent-adolescent education expectations.

**Figure 2:**
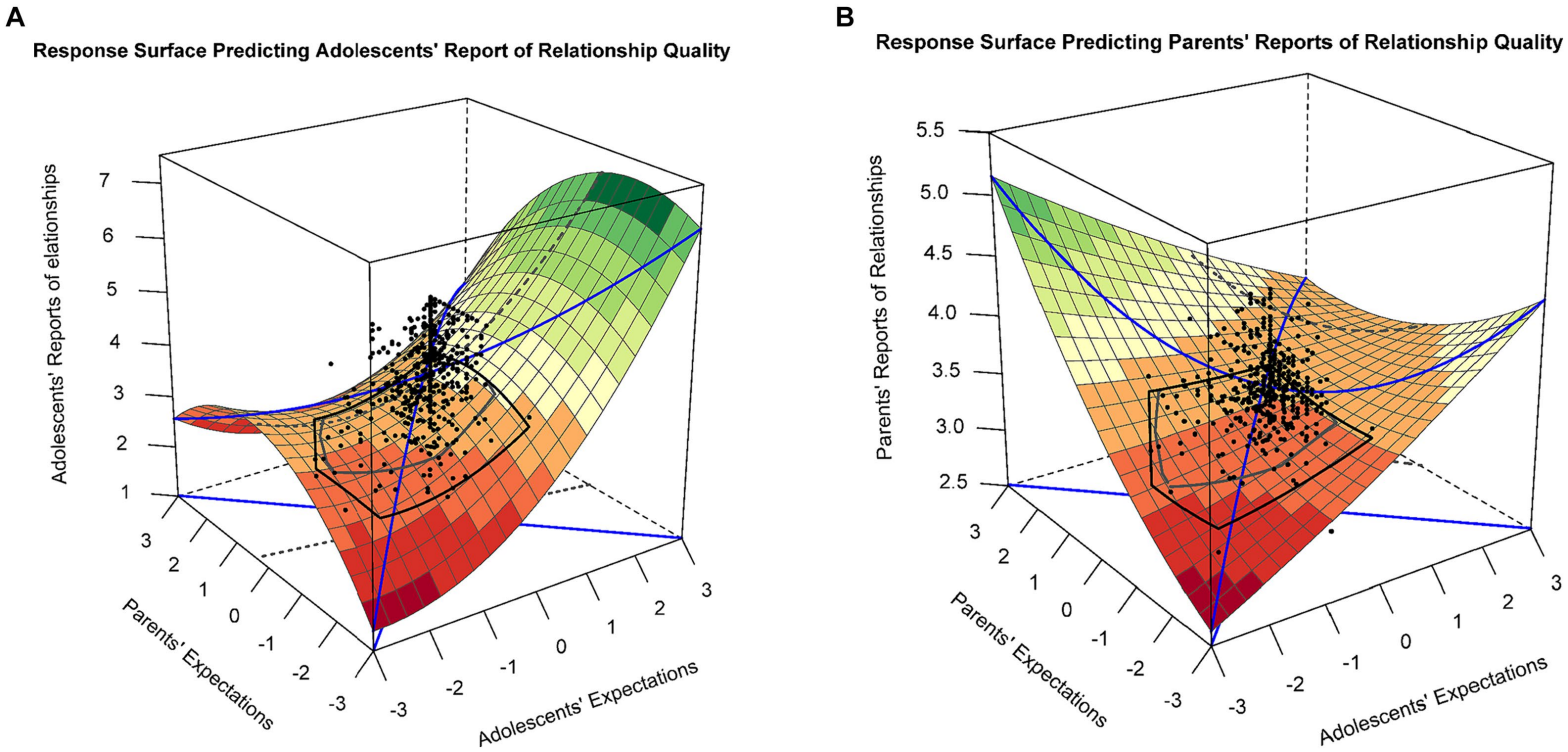
**(A)** Response surface for the polynomial regression of educational expectations predicting adolescents’ reports of parent–child relationships. **(B)** Response surface for the polynomial regression of educational expectations predicting parents’ reports of parent–child relationships.

The polynomial regression of the parent–child relationship reported by adolescents on discrepancies in parent–child educational expectations was included in model 1 of Table 2. The response surface analysis results showed a significant slope along the line of congruence (LOC) [a1 = 0.40, 95% CI (0.149, 0.656), *p* = 0.002] and a nonsignificant curvature along LOC [a2 = −0.14, 95% CI (−0.391, 0.120), *p* = 0.299], indicating a linear additive effect of the surface along LOC. These outcomes suggest that when adolescents and parents report higher educational expectations, adolescents tend to report higher levels of parent–child relationships. Additionally, the slope along the line of incongruence (LOIC) was positive and statistically significant [a3 = 0.70, 95% CI (0.429, 0.964), *p* < 0.01], indicating a linear effect of the LOIC. Specifically, adolescents who reported higher educational expectations than their parents tended to have better quality parent–child relationships (see Figure 2A). The curvature of the LOIC was not statistically significant [a4 = 0.10, 95% CI (−0.236, 0.428), *p* = 0.570].

**Table 2:** Polynomial regression coefficients and response surface parameters.

	Relationship quality		Learning engagement		
	Model 1	Model 2	Model 3	Model 4	Model 5
Polynomial regression coefficients					
b_1_-adolescent report	0.55^***^	0.03	1.17^***^	0.81^***^	1.16^***^
b_2_-parents report	−0.15	0.15^***^	−0.04	0.06	−0.08
b_3_- adolescent report^2^	0.15^*^	0.01	0.03	−0.08	0.02
b_4_- adolescent report × parents report	−0.12	−0.09^*^	0.05	0.13	0.07
b_5_- parents report^2^	−0.17^*^	0.03	−0.15	−0.03	−0.16
b_6_-relationship (adolescents/parents)				0.66^***^	0.26
*R* ^2^	0.13	0.09	0.29	0.45	0.29
Response surface parameters					
a_1_-slope along LOC (x = y)	0.40^**^	0.17^***^	1.13^***^	0.86^***^	1.08^***^
a_2_-curvature along LOC (x = y)	−0.14	−0.05	−0.07	0.02	−0.06
a_3_-slop along LOIC (x = −y)	0.70^***^	−0.12^*^	1.21^***^	0.75^***^	1.24^***^
a_4_-curvature along LOIC (x = −y)	0.10	0.14	−0.17	−0.23	−0.20

Grade levels were controlled for throughout the analyses. ^*^p < 0.05; ^**^p < 0.01; ^***^p < 0.001.

The parent–child relationship in Model 4 (b_6_) was from adolescents’ reports, and the parent–child relationship in Model 5 (b_6_) was from parents’ reports.

The polynomial regression of the parent-reported parent–child relationship on discrepancies in parent–child educational expectations was included in model 2 of Table 2. There was a significant slope along the LOC [a1 = 0.17, 95% CI (0.080, 0.267), *p* < 0.001] and a nonsignificant curvature along LOC [a2 = −0.05, 95% CI (−0.145, 0.051), *p* = 0.345], indicating a linear additive effect of the surface along LOC. These outcomes imply that parents tend to report higher levels of parent–child relationship when both adolescents and parents report higher educational expectations. Moreover, the slope along the LOIC was negative and statistically significant [a3 = −0.12, 95% CI (−0.227, −0.012), *p* = 0.030], indicating a linear effect of the LOIC. Thus, parents who reported higher educational expectations than their children tended to have higher-quality parent–child relationships. The curvature of the LOIC was marginally significant [a4 = 0.14, 95% CI (−0.004, 0.277), *p* = 0.056], suggesting that whether adolescents or parents hold high educational expectations is related to better parent–child relationships as reported by parents (see Figure 2B).

**Figure 3:**
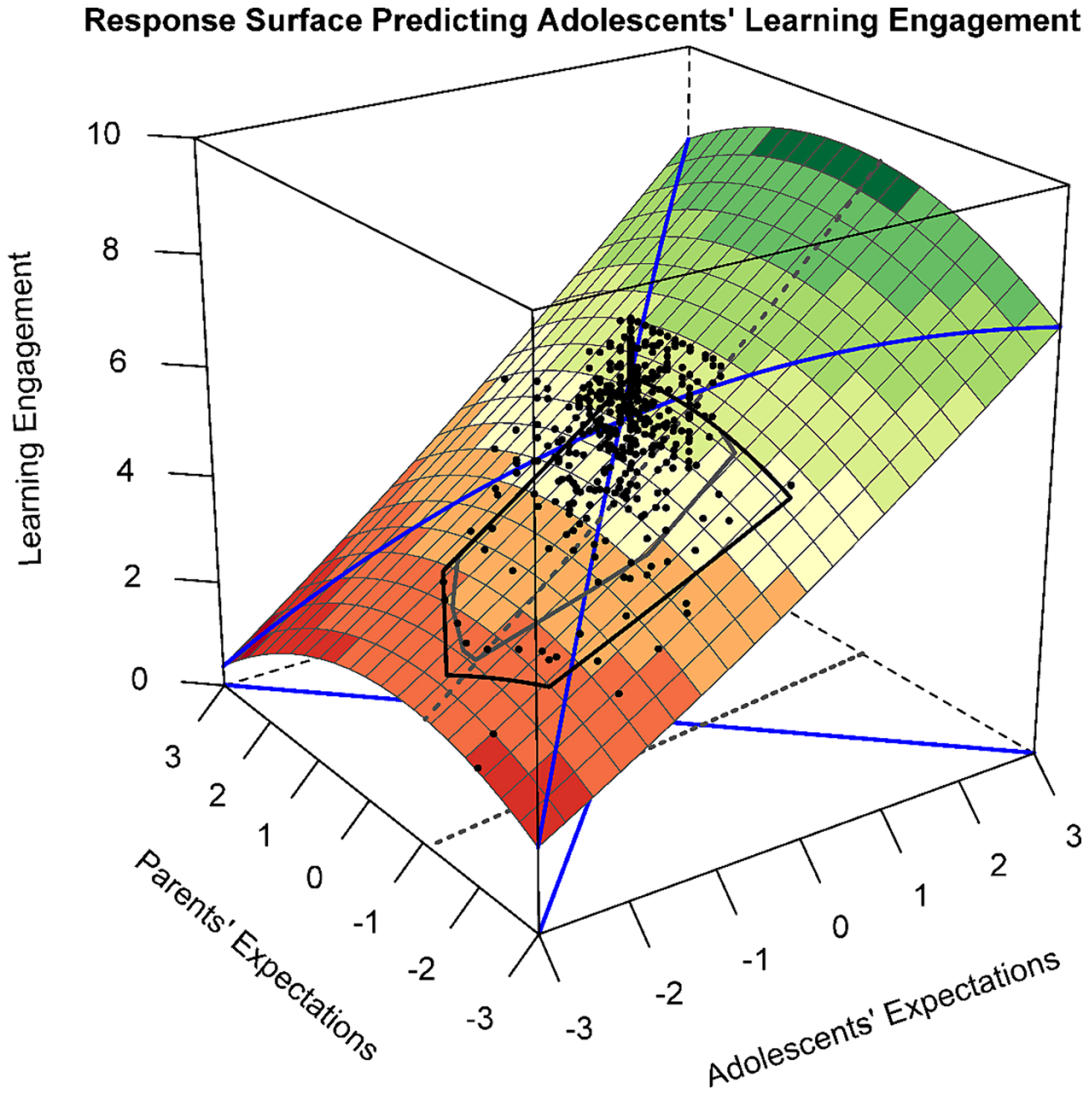
Response surface for the polynomial regression of educational expectations predicting adolescents’ study engagement.

Similarly, in Model 3 (Table 2), the polynomial regression of study engagement on discrepancies in parent–child educational expectations was examined. The slope along LOC was significant [a1 = 1.13, 95% CI (0.778, 1.476), *p* < 0.001], indicating a linear additive effect of the surface along LOC, while the curvature along LOC was nonsignificant [a2 = −0.07, 95% CI (−0.465, 0.322), *p* = 0.721]. Thus, adolescents were more likely to report higher levels of learning engagement when adolescents and parents had higher educational expectations. The slope along LOIC was statistically significant [a3 = 1.21, 95% CI (0.771, 1.652), *p* < 0.001], whereas the curvature was not statistically significant [a4 = −0.17, 95% CI (−0.729, 0.392), *p* = 0.555]. These outcomes suggest that adolescents with higher educational expectations than their parents reported higher levels of learning engagement (see Figure 3).

### 3.3 Testing the mediating effect of parent–child relationship

Substituting Model 1 into Model 4 yielded an equation for testing the mediating effects of the adolescent-reported parent–child relationship and a response surface plot of the mediating effect. Similarly, substituting Model 2 with Model 5 yielded an equation for testing the mediating effects of the parent-reported parent–child relationship and a corresponding response surface plot of the mediating effect.

**Figure 4:**
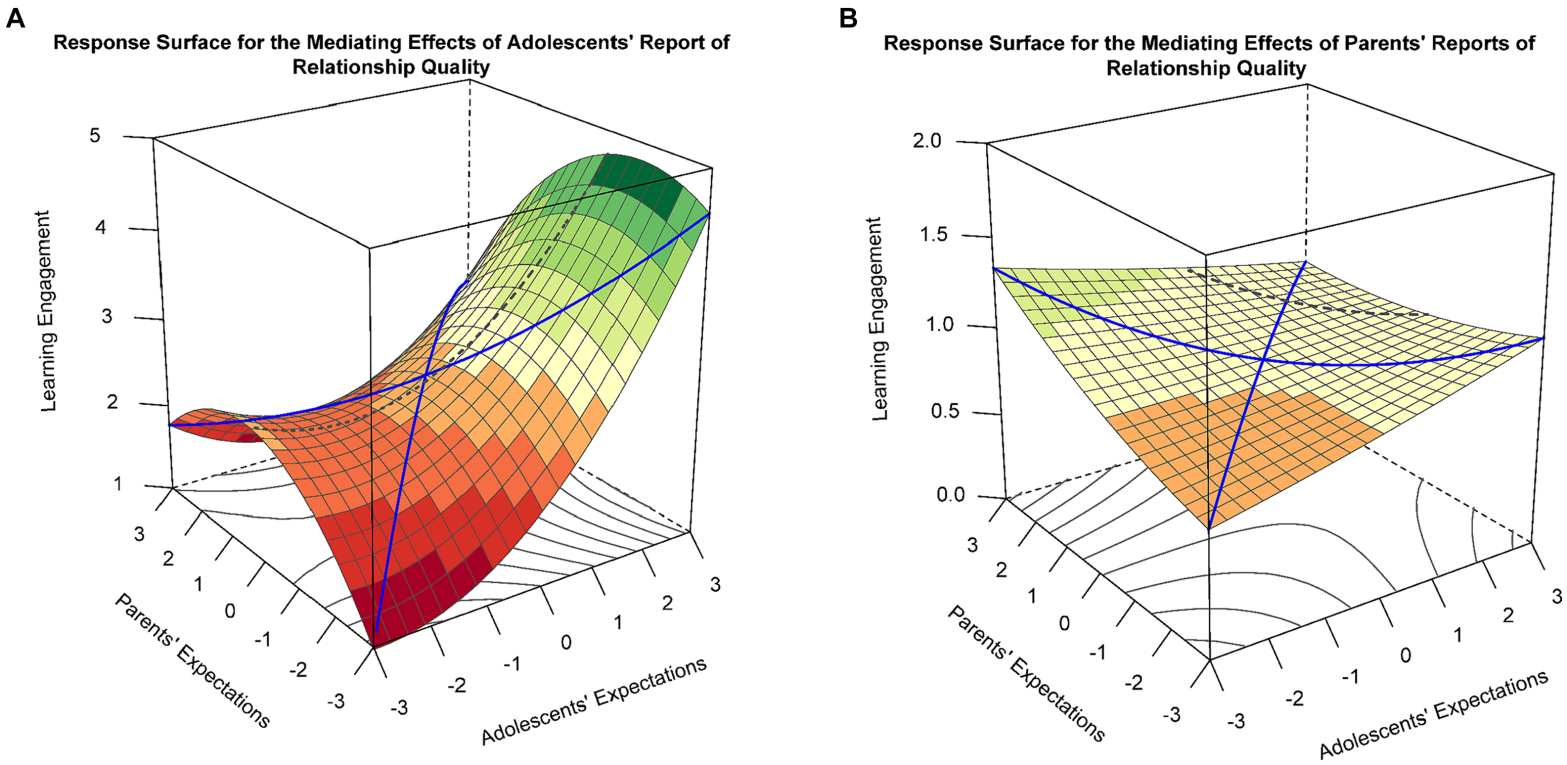
**(A)** Response surface for the mediating effects of adolescents’ reports of parent–child relationships. **(B)** Response surface for the mediating effects of parents’ reports of parent–child relationships.

For the response surface analyses of the mediating effects of the adolescent-reported parent–child relationship (see Figure 4A), there was a significant slope along LOC [a1 = 0.27, 95% CI (0.092, 0.441), *p* = 0.003] and a nonsignificant curvature along LOC [a2 = −0.09, 95% CI (−0.283, 0.104), *p* = 0.363], indicating a linear additive effect of the surface along LOC. That is, high educational expectations could further promote adolescents’ engagement in learning by improving their positive perceptions of the parent–child relationship when both adolescents and parents hold high educational expectations. Furthermore, the slope along the LOIC was positive and statistically significant [a3 = 0.46, 95% CI (0.257, 0.665), *p* < 0.001], but the curvature was not significant [a4 = 0.06, 95% CI (−0.214, 0.342), *p* = 0.653]. Thus, evidence for a linear effect of the LOIC was obtained, indicating that adolescents with higher educational expectations than their parents reported higher satisfaction with the parent–child relationship, which, in turn, facilitated their learning engagement.

For the response surface analyses of the mediating effects of the parent-reported parent–child relationship, neither the slope nor curvature along LOC was significant [a1 = 0.04, 95% CI (−0.019, 0.108), *p* = 0.173; a2 = −0.01, 95% CI (−0.044, 0.020), *p* = 0.460], nor were they significant along LOIC [a3 = −0.03, 95% CI (−0.079, 0.018), *p* = 0.221; a4 = 0.03, 95% CI (−0.026, 0.096), *p* = 0.264]. Additionally, the mediated effect response surface showed a nearly horizontal plane in the three-dimensional coordinate system (see Figure 4B). These results indicated that the parent’s perception of the parent–child relationship did not significantly mediate the relationship between the discrepancies in parent–child educational expectations and study engagement.

We utilized the “block variable” approach to test for mediating effects to ensure the results’ robustness. Path analyses revealed that the block variable, which was derived from the discrepancies between parent–child educational expectations, was a significant predictor of adolescent-reported relationship quality (*a* = 0.32, *p* < 0.001), which, in turn, predicted higher levels of study engagement (*b* = 0.44, *p* < 0.001). The indirect effect of adolescent-reported relationship quality was significant [*ab* = 0.14, 95% CI (0.10, 0.19)], accounting for 29.16% of the total effect. Conversely, the block variable was also found to predict parent-reported relationship quality (*a* = 0.27, *p* < 0.001); however, relationship quality was not predictive of study engagement (*b* = 0.08, *p* = 0.105). The indirect effect was insignificant [*ab* = 0.02, 95% CI (0, 0.05)]. The conclusions drawn from the “block variable” tests of mediating effects were consistent with the above.

## 4 Discussion

The concerns of Chinese parents regarding their children’s education and the academic burden faced by adolescents are pressing issues that require attention. In response, the General Office of the State Council of China issued a document titled “Opinions on Further Reducing the Burden of Homework and Off-Campus Training for Students at the Compulsory Education Stage,” which outlines guidelines for creating a favorable educational environment, alleviating parental stress, and promoting the overall growth and development of students. This multi-informant study utilized the self-system model of motivational development, self-determination theory, and the basic psychological needs hypothesis to examine whether the congruence or incongruence between adolescents’ and parents’ educational expectations was related to adolescents’ study engagement. Addressing the significant educational concerns within the Chinese context, our study introduces two main contributions: first, the application of polynomial regression and response surface analysis for a more nuanced examination of the congruence and incongruence between adolescents’ and parents’ educational expectations; second, an in-depth exploration of the internal mediation mechanisms through the lens of parent–child relationships, offering dual-perspective insights that enrich our understanding of educational dynamics.

Traditional approaches to examining discrepancies in educational expectations have relied heavily on difference scores (Wang and Benner, 2014; Lv et al., 2018), which, despite their utility, present several limitations such as reduced dimensional complexity and interpretational challenges (Shanock et al., 2010). This study advances the methodological framework by employing polynomial regression and response surface analysis, an innovative approach that not only addresses these limitations but also offers a more nuanced understanding of the congruence and incongruence between adolescents’ and parents’ educational expectations (Edwards and Cable, 2009; Shanock et al., 2010; Schönbrodt et al., 2018). By doing so, our research enhances the reliability and interpretability of the findings.

Furthermore, this study meticulously explores the internal mediation mechanisms that underlie the relationship between discrepancies in educational expectations and adolescent academic behaviors. While previous research has touched upon the surface of parent–child educational dynamics (Wang and Benner, 2014), our investigation delves deeper into the mediating role of parent–child relationships, offering insights from both parents’ and adolescents’ perspectives. This dual-perspective approach, grounded in robust theoretical frameworks such as the self-system model of motivational development and self-determination theory, underscores the complexity of educational expectations within the family context. It reveals how these dynamics contribute to adolescents’ learning engagement, thereby providing a comprehensive understanding of the factors that correlate educational outcomes.

### 4.1 Parent-adolescent discrepancies in educational expectations and study engagement

Our study found that high congruence between adolescents’ and parents’ reports of educational expectations resulted in greater engagement in learning for adolescents compared to low congruence. Specifically, higher levels of learning engagement were reported when both adolescents and parents held higher educational expectations. This result supports the study’s Hypothesis 1. Parents with higher educational expectations tend to be more involved in their children’s education, including activities such as parenting, tutoring, and participating in school decisions (Lin et al., 2009; Li et al., 2018). When adolescents hold similar educational expectations to their parents, these expectations can serve as a motivational boost, leading to a synergistic effect that promotes their commitment to learning. Conversely, when both adolescents and parents hold low educational expectations, adolescents tend to have low academic motivation and are least engaged in learning.

The study found that adolescents with higher educational expectations than their parents reported increased levels of learning engagement, supporting Hypothesis 3 and highlighting an incongruence effect. These findings are consistent with previous research showing that parents’ overly high educational expectations can lead to stress for their children (Tan and Yates, 2011; Peleg et al., 2016; Ma et al., 2018). Academic-related stress is a significant issue for adolescents, particularly in China, where traditional Confucian culture is highly valued (Tan and Yates, 2011). Confucian culture emphasizes academic excellence as a form of filial duty that brings honor to the family, while academic failure is viewed as a source of familial shame. To avoid disappointing their parents, adolescents may exert great efforts to meet their parents’ high educational expectations, leading to long-term academic pressure and adverse outcomes such as declining grades, test anxiety, burnout, boredom, and even dropping out of school (Peleg et al., 2016; Ribeiro et al., 2018; Pascoe et al., 2020).

From the perspectives of the self-system model of motivational development and self-determination theory, adolescents’ social interactions with their parents influence their engagement in learning by contributing to adolescents’ perceptions about themselves, which are organized around three basic psychological needs: competence, autonomy, and relatedness (Ryan and Deci, 2000; Skinner et al., 2008). Therefore, adolescents’ needs for autonomy and competence may be thwarted when their parents’ educational expectations exceed their own; further, they experience lower academic motivation (Ryan and Deci, 2000; Wu et al., 2018). In such cases, parents’ heavy investment in their children’s education may not be beneficial, as it is more likely to result in a drag effect. Conversely, when adolescents’ educational expectations surpass their parents’, they are more academically autonomous and experience higher internal motivation, leading to a stronger focus and persistence in their studies, as well as a greater sense of joy and value in the learning process. Additionally, children who exceed their parents’ educational expectations are a source of pride for their parents, which can lead to increased praise and recognition, further strengthening their academic engagement. These findings suggest that adolescents’ own educational expectations are the primary intrinsic driver for promoting engagement in learning. Whether parental educational expectations exert a positive influence on study engagement depends on the adolescents’ educational expectations, with those who have higher expectations consistently favoring enhanced engagement in their studies.

### 4.2 The mediating roles of parent–child relationship

The current study found evidence for the congruent effects of educational expectations in predicting the parent–child relationship reported by adolescents and parents. Specifically, in families where both adolescents and parents reported high educational expectations, they were more likely to report good relationships. This result supports Hypothesis 2 of the study. The high level of congruence in educational expectations between adolescents and parents suggested that their shared high educational expectations may synergistically promote adolescents’ academic development and positive social adjustment. In contrast, when both parties had low educational expectations, it was detrimental to maintaining a good parent–child relationship. This may be due to the lack of emotional bonding between parents and adolescents in families with low educational expectations, where parents may be more permissive and hands-off in their parenting style (Wu and Yang, 2012).

Interestingly, when there were discrepant educational expectations between adolescents and parents, their reports of the parent–child relationship were quite different. Adolescents reported better parent–child relationships when their educational expectations were higher than their parents’ expectations, while the opposite was true for parent-reported parent–child relationships. This result supports Hypotheses 3 and 4 proposed in this study regarding the incongruent effects of parent–child relationships on parental educational expectations. This finding aligns with previous research on adolescent-parent discrepancies in views of the parent–child relationship (Nelemans et al., 2016). Both parents and adolescents held their own views on the parent–child relationship. Adolescents viewed gaining more autonomy in school and receiving parental praise as positive aspects of a good parent–child relationship. Specifically, adolescents’ higher educational expectations than their parents contributed to greater autonomy, which satisfied their need for autonomy according to self-determination theory (Ryan and Deci, 2000). The satisfaction of autonomy brings more positive emotions, which can broaden and build resources that help adolescents build a solid family support system and promote good parent–child relationships (Fredrickson, 2001). In addition, adolescents with high educational expectations are often perceived as more enterprising and ambitious, which may make them more likely to be favored by their parents. Conversely, when adolescents’ educational expectations are lower than their parents’, parents’ excessive educational expectations may become a trigger for parent–child conflict and damage the parent–child relationship (Chiang and Ellis, 2019). In contrast, parents viewed a good parent–child relationship as the responsibility of training children and having them achieve success in terms of academic performance. This interpretation is further confirmed by the non-linear effect along the line of incongruence. When either adolescents or parents hold higher educational expectations than the other party, it is associated with better parent–child relationships reported by parents. This result suggests that, from parents’ perspective, parents may be satisfied either when they themselves hold higher educational expectations or when they have an enterprising child.

It should be noted that the results of the mediating effect test for parent–child relationships revealed that child-reported, but not parent-reported, parent–child relationships significantly mediated the association between discrepancies in parent–child educational expectations and study engagement. This result supports Hypothesis 5 proposed in this study. This finding aligns with previous research indicating that adolescents’ perceptions of the family environment are a stronger predictor of their adjustment than parents’ perceptions (Human et al., 2016). Additionally, our findings confirm that adolescent-reported parent–child relationships are more likely to be significant family social resources, substantially facilitating learning engagement.

### 4.3 Implications

The present study investigated the association between parent–child discrepancies in educational expectations and study engagement among adolescents from both the parents’ and adolescents’ perspectives. Additionally, the study explored the mediating roles of parent-reported and adolescent-reported parent–child relationships. The response surface analysis approach was utilized to graphically and insightfully reveal the relationship between different matches of parent–child educational expectations and adolescent learning engagement, along with its internal mediating mechanisms. The study’s findings not only enhance the understanding of how parent–child discrepancies in educational expectations are linked to adolescents’ learning engagement but also provide targeted recommendations for parents and educational practitioners to promote youth engagement in learning.

The present study demonstrated that higher parental educational expectations may not always lead to greater academic engagement in children but rather depend on the level of adolescents’ educational expectations. Parental expectations are more likely to motivate adolescents when their expectations are comparable to or higher than their parents’. Although adolescents tend to be more engaged in learning when their educational expectations exceed those of their parents, this advantage may be limited in the long term due to the potential lack of parental involvement and support when parents have lower educational expectations (Li et al., 2018). Therefore, it is preferable for both parents and adolescents to hold high and comparable educational expectations.

Moreover, attention should be given to the perceived functional differences in the parent–child relationship between parents and adolescents. The study found that adolescent-reported parent–child relationships could mediate the link between parent–child discrepancies in educational expectations and study engagement, whereas parent-reported parent–child relationships did not. Adolescents may require more autonomy and self-awareness, which may lead to a more complex set of needs that differ from what their parents perceive as a good parent–child relationship (Eccles et al., 1993). Discrepancies between parents’ and adolescents’ perceptions of parent–child relationships have been linked to adolescents’ internalizing and externalizing problems (Ohannessian, 2012; Ohannessian and De Los Reyes, 2014; Nelemans et al., 2016). This suggests that parents should be more empathetic in their child-rearing practices and pay closer attention to their children’s wants and desires. To enhance children’s engagement in learning, parents should provide more autonomy support that encourages the child’s intrinsic motivation (Froiland and Worrell, 2017).

### 4.4 Limitations and future lines of research

Building on the insights from our study, there are several avenues for future research that merit attention. Firstly, due to the cross-sectional nature of our research, we acknowledge that establishing causality between parental expectations, adolescent study engagement, and relational qualities remains a challenge. Longitudinal studies are thus essential to decipher the directional influences and temporal changes in these relationships over time.

Secondly, our study’s focus on a Chinese context raises questions about the universality of our findings. Given the documented higher educational pressures faced by students in China and other Asian countries compared to their Western counterparts (Tan and Yates, 2011), it is crucial for future research to investigate whether these patterns hold true across different cultural and educational landscapes. Comparative studies across diverse cultural settings could illuminate the interplay between cultural norms, parental expectations, and adolescent academic outcomes, providing a more nuanced understanding of these dynamics.

Thirdly, the predominance of data from mothers in our study, as opposed to fathers, presents a unique lens through which we viewed our findings. Although our analyses suggest that the inclusion of data from both parents does not significantly alter the results, literature indicates that mothers and fathers may engage differently in their children’s education (Phares et al., 2009). Future research could benefit from delving deeper into these differences, exploring how maternal and paternal roles distinctly influence adolescents’ educational experiences and outcomes. This could involve qualitative approaches to capture the depth of parental involvement or quantitative measures to assess the impact of each parent’s expectations on adolescent well-being and academic engagement.

Moreover, additional variables such as adolescent self-esteem, parental styles, and the educational environment could provide further insights into the mechanisms through which parental expectations affect adolescent development. Investigating these factors would not only enrich our understanding of the parent-adolescent dynamic but also offer actionable insights for educational practitioners and policymakers aiming to foster environments that support healthy academic motivation and engagement.

## 5 Conclusion

This study provides evidence for associations between parent-adolescent discrepancies in educational expectations and study engagement, as well as adolescent- and parent-reported parent–child relationships. Specifically, the congruent effects of educational expectations between parents and adolescents suggest that adolescents demonstrated higher levels of engagement in learning when both adolescents and parents reported congruent and higher educational expectations. Furthermore, higher quality parent–child relationships were reported by both parents and adolescents when both adolescents and parents reported congruent and higher educational expectations. Second, the incongruent effects of educational expectations between parents and adolescents suggest that when adolescents held higher levels of educational expectations than what was reported by their parents, they were notably more likely to report higher levels of engagement in learning and better parent–child relationships. Conversely, parents reported better relationship quality when they held higher educational expectations than their adolescents. Lastly, the association between discrepancies in expectations and study engagement was significantly mediated by adolescent-reported relationships but not parent-reported ones.

## Data availability statement

The raw data supporting the conclusions of this article will be made available by the authors, without undue reservation.

## Ethics statement

The studies involving humans were approved by Central China Normal University, Ethic Committee, EC, Institutional Review Board. The studies were conducted in accordance with the local legislation and institutional requirements. Written informed consent for participation in this study was provided by the participants’ legal guardians/next of kin.

## Author contributions

YS: Conceptualization, Formal analysis, Methodology, Software, Visualization, Writing – original draft, Writing – review & editing. JW: Conceptualization, Data curation, Investigation, Resources, Writing – review & editing. ZZ: Funding acquisition, Project administration, Supervision, Validation, Writing – review & editing. YT: Funding acquisition, Supervision, Validation, Writing – review & editing, Project administration. WL: Methodology, Writing – review & editing. HX: Methodology, Writing – review & editing.
